# Stigmatisation experiences in families with hereditary conditions: an exploratory study

**DOI:** 10.1007/s12687-025-00784-5

**Published:** 2025-03-25

**Authors:** Joana Oliveira, Álvaro Mendes, Milena Paneque

**Affiliations:** 1 FPCEUC - Faculty of Psychology and Educational Sciences, University of Coimbra, Coimbra, Portugal; 2 CGPP - Centre for Predictive and Preventive Genetics, IBMC - Institute for Molecular and Cell Biology, University of Porto, Porto, Portugal; 3 i3S - Institute for Research and Innovation in Health, University of Porto, Porto, Portugal; 4 ICBAS - School of Medicine and Biomedical Sciences, University of Porto, Porto, Portugal

**Keywords:** Genetic disease, Stigma, Psychosocial impacts, Coping strategies, Discrimination

## Abstract

Hereditary conditions can pose several challenges to the individual and their family members. In addition to the symptoms of the condition itself, stigmatisation is often described by those who live with hereditary conditions as a major challenge. This study explores the stigmatisation experiences of people with inherited conditions and their families in Portugal. Seventeen semi-structured interviews were conducted with individuals affected with a hereditary condition, asymptomatic carriers and family members, recruited through patient support organizations and social media. The data were analysed through inductive content analysis, resulting in three major categories: (i) stigmatisation contexts; (ii) psychosocial impacts; and (iii) coping strategies to deal with the stigma. The findings suggest the perception of stigma in family and social life, including specific contexts and systems such as academic, work, health care, social security and insurance. The stigma is associated with embarrassment, sadness, and frustration at the personal level, and with social impacts such as isolation, interpersonal distance, and avoidance of relationships. Participants often resort to providing explanations about their condition and to social isolation as a coping strategy for dealing with stigma. This study provides insights that reinforce the continuous need to raise awareness about hereditary conditions at a societal level and their associated impacts, to provide specific training for healthcare professionals on the potential stigma attached to inherited conditions, and to implement national strategies to reduce stigmatisation.

## Introduction

Hereditary conditions are characterised by the possibility of being passed down from generation to generation through chromosomal alterations or in the genes themselves. Depending on the mode of transmission—autosomal recessive, autosomal dominant, X-linked, mitochondrial or multifactorial—individuals can inherit, transmit and manifest these conditions in different ways (Korf 2004; Tobias et al. 2011). In autosomal recessive transmission, the individual develops the hereditary condition only if they inherit a pathogenic variant from both parents (Korf 2004). In autosomal dominant transmission, the individual can develop the hereditary condition even if only one parent carries the pathogenic variant (Korf 2004). In X-linked transmission, males are more likely to develop the condition, while females are usually carriers and rarely exhibit symptoms (Korf 2004). In mitochondrial transmission, all individuals with a mother carrying the pathogenic variant can develop the condition, but only women can transmit it to their offspring (Tobias et al. 2011). Finally, in multifactorial transmission, the development of the hereditary condition depends on genetic factors (polygenes) and environmental influences, making it more difficult to predict (Tobias et al. 2011). Hereditary conditions often involve physical, cognitive and/or emotional symptoms that vary depending on the condition. And in addition to the clinical challenges, hereditary conditions bring significant social and emotional consequences. Families need to adapt to the changes imposed by the condition, integrate clinical information and deal with the psychological and social impacts (Street and Soldan 1998). The diagnosis can generate feelings of anger, disappointment, disbelief, displacement, pain, shame and guilt, as well as interfering with relationships and the decision to have children (Ragusa et al. 2020; Street and Soldan 1998). Although it is possible to carry out predictive tests whose results make it possible to predict whether condition will develop in the future, the decision to carry them out is also complex and involves various psychosocial issues (Mendes et al. 2019; Rolim et al. 2006). In addition to these emotional impacts, hereditary condition symptoms often restrict social and work activities, leading to economic difficulties and barriers to community integration (Faure et al. 2019; Ragusa et al. 2020; van der Zwaan et al. 2022; Zorcec et al. 2019). Experiences of stigma in people living with these inherited conditions have also been described recently (Foji et al. 2021; Fournier et al. 2023; Ragusa et al. 2020; Sherman et al. 2020).

According to Goffman (1963 as cited in Major and O’Brien 2004), the first author to define stigma, it occurs when a person has an attribute that is seen as undesirable in the eyes of society in general and, as a result, society in general begins to define them based on this attribute and to depreciate them as a whole. Link and Phelan (2003) defined stigma as a combination of co-occurring components: labelling differences; negative stereotypes; separation; status loss and discrimination; dependence of stigma on power. According to the authors, people begin by labelling and distinguishing human differences (labelling differences); through dominant cultural beliefs they make these differences undesirable characteristics, and associate the labelled individual with this characteristic (negative stereotypes); people with these undesirable characteristics are placed in a different category from the rest of society (separation); with this separation into two groups, people can experience discrimination, which is the concrete action of stigmatisation, for example through exclusion, rejection and devaluation (discrimination); discrimination can be enacted from one individual to another or through social and structural inequalities and it can result in status loss, which culminates in loss of social, economic and political power (status loss and dependence of stigma on power) (Abbey et al. 2011; Link and Phelan 2003). Stigmatisation restricts access to important areas of life and has a direct influence on the social status, psychological well-being and physical health of the discriminated group (Major and O’Brien 2004). Kane et al. (2019) describe different types of stigma: perceived stigma, anticipated stigma, internalized stigma and experienced/enacted stigma. Perceived stigma refers to the interpretation of certain attitudes or behaviours as markers of difference. Anticipated stigma refers to the fear of future stigmatisation. Internalised stigma refers to self-stigma, in which the stigmatised person accepts and applies the stigma to themselves. Finally, enacted stigma refers to concrete actions of discrimination.

Studies highlight that individuals with hereditary conditions face different types of stigmatisation in different contexts. In the family context, a diagnosis of IC can trigger negative reactions, including shame, guilt and interpersonal conflicts, especially when there is a risk of hereditary transmission (Manz 2016). In the community there are several reports of social rejection, social exclusion and labelling, namely due to a lack of understanding of the symptoms and the disease (Mendes et al. 2017; Sherman et al. 2020; Ragusa et al. 2020). Individuals describe difficulties in maintaining friendships and romantic relationships (Mendes et al. 2017; Ragusa et al. 2020). In the context of employment, individuals describe fear of being penalised for the knowledge that they have an inherited condition (van der Zwaan et al. 2022) and report that their superiors don't understand why they have to miss work to go to appointments and why they can't perform certain tasks in the workplace (Faure et al. 2019). In the school context, there are reports of children being rejected by their peers and feeling treated differently by teachers (Aghaei et al. 2023; Ragusa et al. 2020). In the health services, experiences have been described that mention the tendency of doctors to attribute any symptom to hereditary condition, ignoring potential alternative diagnoses, as well as a lack of empathy and preparation by some health professionals and difficulties in getting access to the necessary therapies (Costa et al. 2022; Etchegary 2007). And in insurance companies, there is a fear that insurance contracts will be jeopardised or even denied due to the presence of a hereditary condition (Manz 2016; Otlowski et al. 2012). These experiences of stigma have a deep psychological impact on the people who live them. Stress, embarrassment (Etchegary 2007), anxiety (Manz 2016), shame and sadness (Mendes et al. 2017) are some of the feelings triggered by stigma. Fear is always very present, specifically: of being stigmatised, of their children being discriminated against, of losing their jobs and of losing their life and health insurance because they have an inherited condition (Manz 2016; Otlowski et al. 2012; van der Zwaan et al. 2022). Often, as a consequence of these emotions and feelings arising from stigmatisation, people with hereditary conditions isolate themselves (Manz 2016). And at the same time, they feel guilty for losing people due to their own isolation (Mendes et al. 2017). The lack of support and understanding contributes to a cycle of exclusion, which affects not only the individuals directly affected, but also their family members, who often reports feeling frustrated, displaced, discredited, angry and see their social life threatened, also experiencing isolation (Ragusa et al. 2020).

The studies that explore the stigma experiences of individuals living with hereditary conditions tend to focus only on one hereditary condition or only touch briefly on the experiences of stigma, not only in Portugal but internationally. As far as we know, this is the first study to specifically explore the experiences of stigmatisation in those affected by hereditary conditions in a broad way. This study aims to explore and map the experiences of stigmatisation of individuals with hereditary conditions and their families in Portugal, specifically: i) in social, relational, work, healthcare, social security and insurance contexts; ii) how stigmatisation occurs and who instigates it; iii) the emotional and social impacts of stigmatisation; iv) to understand how people cope with stigma and what resources they draw upon to confront it.

## Method

### Study design

This research followed a qualitative and exploratory approach by conducting semi-structured interviews to explore and map the experiences of stigmatisation of individuals with hereditary conditions and their families in Portugal. This study was approved by the Committee for Ethical and Responsible Conduct of Research of the Institute for Research and Innovation in Health (i3S).

### Participants

The participants were recruited through patient support organizations and social media. An invitation was addressed to RD Portugal, an umbrella association with more than 40 Portuguese associations that represent these conditions, to share the study with associations that support families with hereditary conditions. Subsequently, an e-mail was sent to each of the associations to invite them to spread the study among its members. The 19 associations which accepted the invitation shared the study in their social media and/or internally among their members. The disclosure contained all the relevant information about the study, i.e., study aims, inclusion criteria, how they could participate, the ethical committee approval and the study leaders. The disclosure includes a link and a QR Code, which gave access to a form where the participants could fill their email, if they wanted to participate in the study. The participants who completed and returned the form where contacted, by e-mail, with study indications and available schedules to conduct the interview. Once the schedule was selected, a link was sent to carry out the interview, via Microsoft Teams. The inclusion criteria were: (i) individuals affected by a hereditary condition, or carriers of a hereditary condition, or biological or non-biological family members of individuals with hereditary condition; (ii) have age equal or higher than 18 years and be fluent in Portuguese.

Thirty-nine individuals indicated interest in participating and 18 of them scheduled the interview. Verbal informed consent was obtained from all participants instead of written informed consent. This decision was related to fact that the interviews were conducted remotely and the use of hard copies of the consent form would potentially jeopardize data protection and privacy. The informed consent form was read out by the interviewer at the beginning of the interview and the participants were asked whether they agree to the study and wished to continue or not. The participants had previously expressed their interest in to participate in the study and had the chance to clarify any doubts. Seventeen interviews were conducted (participation rate: 43.6%). The sociodemographic data is described in Table 1 and the hereditary conditions present in the study are given in Table 2.

**Table 1:** Participants’ sociodemographic information

Sociodemographic characteristics	N	%
Sex		
Female	14	82
Male	3	18
Marital status		
Single	5	29
Married/ Common law	10	59
Separated/ Divorced	1	6
Other	1	6
With children		
Yes	11	65
No	6	35
Condition Status		
Affected	9	53
Asymptomatic carrier	1	6
Not affected	7	41
Patient support organization member		
Yes	13	76
No	4	24

**Table 2:** Hereditary Conditions present in the study families

Hereditary condition in the family	N
Huntington Disease	5
Hereditary Ataxias	4
Hereditary Psoriasis	2
Skeletal Dysplasia	1
Neurofibromatosis	1
Non-Obstructive Hypertrophic Cardiomyopathy	1
Congenital Central Hypoventilation Syndrome (CCHS)	1
Amyotrophic Lateral Sclerosis (ALS)	1
Familial Amyloidotic Polyneuropathy (FAP)	1

### Procedure

Based on a review of the relevant literature a topic guide was developed and then questions were devised to cover them. To ensure the content validity and increase the quality of the data collected in the future an invite to researchers in the field, medical geneticists, psychologists and members of the patient support organizations was addressed by e-mail to evaluate the relevance, sensitivity and clarity of the questions (Allen et al. 2023). The feedback from 6 professionals was integrated (2 psychologists, 2 medical geneticists, 1 genetic counsellor and 1 patient association representative). Then, to verify the face validity, the interview guide was presented to individuals affected by a hereditary condition, carriers of a hereditary condition and family members. They evaluated the clarity, relevance and sensitivity of the questions, one by one, and were questioned about the comprehension of each question and whether the interview guide captured the overall experience of living with a hereditary condition (Allen et al. 2023). Finally, the suggestions of the 4 participants (2 affected, 1 carrier and 1 family member) have been incorporated into the interview guide.

Semi-structured interviews were conducted by videocall on Microsoft Teams by JO from February to April 2024. All interviews were recorded with participants’ authorization and then transcribed in European Portuguese. The interviews lasted 20 min to 1 h and 15 min (mean: 41 min). First were collected the sociodemographic information of the participants and then open questions about the experience of living with a hereditary condition were asked (Table 3). The questions did not follow an order, but all were asked of all the participants.

**Table 3:** Semi-structured interview guide

	Questions
Sociodemographic data	1. Can you tell me a bit about yourself (age, marital status, household, involvement with patient support organizations)? 2. What hereditary condition runs in your family? 3. Are you a carrier of a genetic alteration that causes a hereditary condition (with or without symptoms), are you at risk of inheriting the disease, or are you a relative (biological or non-biological) of someone with the disease? If you are a relative, are you a carer for the person with the disease?
Experiences of living with a hereditary condition	4. Now I'm going to ask you to tell me a bit about [the name/expression the patient/family member uses for the condition] and what it's like for you to live with the condition/the experience of living with the condition in the family 5. What was it like (for your family member) deciding to share with others (e.g. family, friends, at work/school/university) that you/they had the disease? When did you/they decide to share? 6. What influence does your (family member's) hereditary condition have on your/their daily life in terms of your routines (e.g. shopping or using public transport), your/their social life and your/their hobbies? 7. Regarding your future prospects, do you have any fears about the progression of your/their condition? What? 8. What impact does [name/expression the patient/family member uses for the illness] have on your/their family relationships (biological and/or non-biological), friendships, love relationships and relationships with work/school/university colleagues? 9. What impact does [name/expression the patient/family member uses for the condition] have/had on decisions you/they have made throughout your/their life—such as getting married, having children and career choices? 10. On a social level (in your community, at work/school/university), what are the biggest challenges/difficulties you face because of your condition? / On a social level (in community, at work/school/university), what are the biggest challenges/difficulties that your familiar face because of their condition? 11. What are the biggest challenges/difficulties you encounter in the context of health services? 12. How are you (How is your family member) treated in health services (e.g. hospitals, health centres, clinics) by the various health professionals (e.g. doctors, nurses, assistants, administrators, security guards)? 13. How are you (how is your family member) treated by the social security services?
Experiences of stigmatisation in individuals with a hereditary condition	14. Have you ever felt that you were discriminated against/witnessed your relative being discriminated against because of [the name/expression the patient/family member uses for the condition]? Can you please describe situations in which this has happened? 15. What do you usually do to deal with these situations? If it's not recurrent, can you describe something you've done to deal with the situation(s)? 16. What have you stopped doing because of the disease/stigma or fear of associated discrimination?

## Data analysis

Inductive content analysis was applied to analyse the data, as this approach is considered appropriate when there is not much literature on the subject and the aim is not only to describe and understand the phenomenon, but also to obtain results that are relevant to practice (Vears and Gillam 2022).

Following the procedures suggested by Vears and Gillam (2022), first all the interview transcripts were read to become familiar with the data. Then the units of meaning were identified and labelled with big-picture category codes in all the interview transcripts, culminating in a preliminary list of categories. The next step was identifying subcategories and to refine the big-picture codes. Then all the subcategories were compared and refined, comparing and relating their content and meaning, until a final coding scheme was obtained. To increase the rigour of the data analysis, the interview transcripts were analysed by three different researchers (JO, ÁM and MP) (FitzPatrick 2019). JO and MP independently read all the transcripts to develop a list of categories and subcategories. Then, both met to discuss their proposals and, when doubts arose, a third researcher (ÁM) was included. This process of refinement was repeated until agreement was reached. Finally, the data was summarised and interpreted in order to create an integrative description.

## Findings

**Fig. 1:**
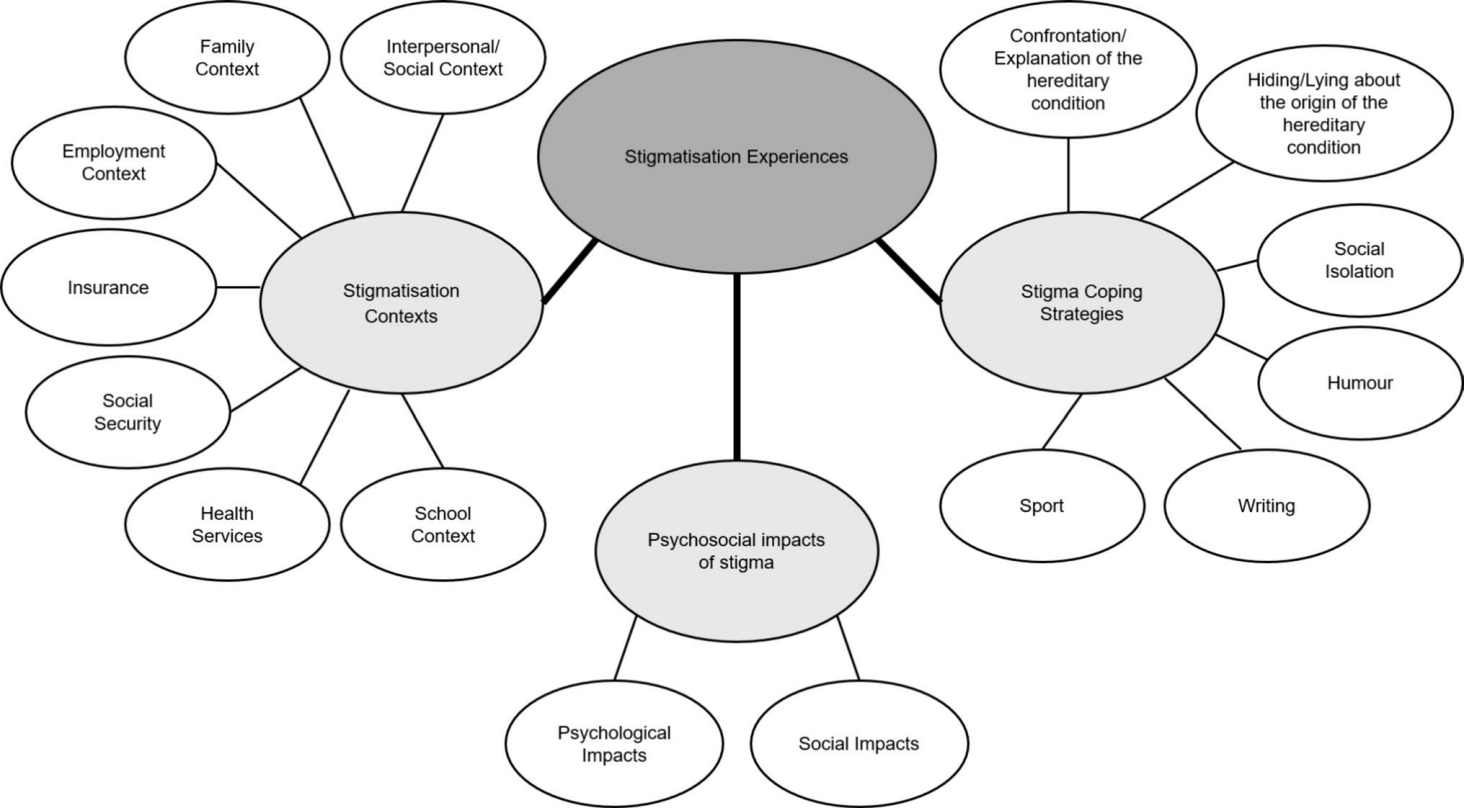
Categories and subcategories resulting from data analysis

Three main categories were identified: (i) stigmatisation contexts; (ii) psychosocial impacts; and (iii) stigma coping strategies. Figure 1 introduces a conceptual map of categories and subcategories identified in the analysis. The categories are not mutually exclusive and not follow any sequence. Each category is presented with excerpts from the interview transcripts to illustrate the key points, followed by a code of the participant with his age, gender and an indication of the hereditary condition status (affected, asymptotic carrier and not affected). Some sociodemographic details of the participants have been changed to preserve the privacy of the participants. “I” refers to interviewer and “P” to participant. The content between square brackets indicates the suppression of details that could compromise participant confidentiality or the introduction of details to make the extract clearer. “(…)” indicates the suppression of parts of the text that are not relevant to the description of the experience of stigmatisation.

## Stigmatisation contexts

This category describes how, by whom and in which places the participants have experienced some type of stigmatization, through the attribution of labels, negatives stereotypes, separation, discrimination, or loss of status. Stigmatisation seems to be present in family, social contexts, at school and at the workplace, in health services, social security and in insurance purchases. In Table 4 we present several excerpts that support each subcategory of the contexts of stigmatisation.

**Table 4:** Supporting quotes for stigmatisation contexts

Subcategories	Excerpts
Family Context	a) “Okay. And no, we don’t get invited very often when it’s something [more important festivities], we’re not. That’s it. My mother-in-law calls us home, sometimes too much, every Sunday. But, for example, if certain people come, no, we don’t go. And sometimes I say to my husband ‘one day I’ll stop going permanently’. (…) But sometimes it’s annoying because so-and-so is here, we don’t get called anymore.” (P5, 53, F, affected by Neurofibromatosis and mother of two affected children) b) "Even by her cousins, she's [P10's daughter] often not invited to family parties where her uncles are (…). Her cousins have parties with [other] cousins and friends and she doesn't go. Even there [in the family context] she is discriminated against." (P10, 55, M, Not Affected, father of someone affected with CCHS) c) “But there are those little things that I sometimes think could have been avoided. It could have been avoided. Making fun of my son at my mother-in-law's house. People who are distant cousins and that one, that one wasn't even a cousin and a sister-in-law of mine, and my mother-in-law not being able [to reprimand them]… My son was 16, 17 at the time and reacted as if he was 10, he watched Noddy [cartoons] and started talking about Noddy to his older cousins. And another one who wasn't even a cousin. And I even said at the time, ‘oh son, no, don't do that’. I called him out on it. I caught a conversation on Facebook… On Facebook, between her, a woman and my sister-in-law asking about Noddy. It was my son… for fun” (P5, 53, F, affected by Neurofibromatosis and mother of two affected children)
Social/ Interpersonal Context	d) “So we in the neighbourhood at the beginning, when my mother [with Huntington disease] walked down the street, always with her imbalances, there was, this, way that people… I’m not going to say worrying about, but they say “whoa, the lady is always drunk in the street, staggering around, is that any way to be?” Then there was the part about the children not taking care of the mother, “They let the mother out on the street”, because we already had notion of the disease. Then, while my mother could walk, we let her, “Go for a walk, mother, go for a walk, go to the park, comes back, we are here, if you need something, call, we’ll come to you”. So, she seemed to be abandoned. Then there was always the social criticism that the family wasn’t taking care of the person.” (P12, 38, F, Not Affected, daughter and sister of individuals affected by Huntington’s disease) e) “Look, I, I can give you examples, look how a very simple thing, being an altar server in church, I was never allowed to be, because of my height, because of my problem. The simple gesture of making the request with the baskets. In my time they wouldn't let me. My son goes now, but I still have to ask them to let my son go.” (P6, 41, F, Affected by Skeletal Dysplasia and mother of a child affected) f) “Yes, I felt discriminated against when I was younger. Yes. For example, the swimming pool thing, right? Being forced to leave a public swimming pool. The stares, even when people start to realise what psoriasis is. Of course, there would still be stares. When you go to the beach, when you go to an outdoor pool. At the pool I used to go to, everyone already knew about it and then there were no more stares. But when I went to a new pool, when I went to have a game of water polo in a new pool, we always felt those stares. Or sometimes the stares wouldn't even be at me, or they would be, but not because of the psoriasis. But the first interpretation was that it was because of the damaged skin, yes.” (P11, 38, F, Affected by Hereditary Psoriasis)
School Context	g) “In school, they always had to be very attentive to her [child with CCHS, a hereditary condition in which a person may suddenly stop breathing due to failures in the automatic control of breathing]. It was always a focus there, always very big, she had all eyes [from staff and teachers], which was not always a good thing, because by children from her age, she was often seen as deficient, sometimes they treated her that way. In this phase, they are… it doesn’t mean that they are cruel, they have not filter. Children are as they are and, there you go, they don’t have those filters with the fear of not hurt, they don’t say it to hurt. They say the things as they see and, even more, she, which had there a lot of attention… and with children which don’t have so much attention at home, often suffered there… some physical violence” (P10, 55, M, Not Affected, father of someone affected with CCHS) h) “Maybe it's because they think we're not capable of doing things (…) and sometimes people try to make life easier for us. They don't let us manage on our own. I've felt that now with my son's teachers. There are two or three teachers who treat him like a poor child, and he doesn't like it. He comes home very angry. And I think it's a bit like that, he sometimes feels excluded.” (P6, 41, F, Affected by Skeletal Dysplasia and mother of a child affected) i) “My son's first years at school were difficult. Because he was very, very childish for his age. Then, when he came to school, he was in fifth grade and played with the kids in tenth grade, because the kids in his class didn't play with him, didn't help him, ignored him, that's it.” (P5, 53, F, affected by Neurofibromatosis and mother of two affected children)
Employment Context	j) “I tried to find another job, but I have always been very honest, I said that I suffered from a neurodegenerative disease, I was confined to a wheelchair. But they ignored a lot, plain and simply. They didn’t say nothing, neither yes, neither no. And I then, I retired due to disability.” (P1, 49, M, affected by Friedreich’s Ataxia) k) “So professionally, of course, it affects everyone else. In the case of my brother, who has now been diagnosed with the disease, there was always a concern from his colleagues, because he's a postman, he drives the post office vans a lot. He has certain tics and a way of being that when he speaks, when you speak to him, he stops for a while and thinks, it takes him a little longer to answer. So there was always the concern and the way… [his colleagues] used to say, “Oh, well, that's it, after lunch, he's already drunk, he's no longer working or he's already arrived drunk”. There was a lot of this observation.” (P12, 38, F, Not Affected, daughter and sister of individuals affected by Huntington’s diseases
Social Security	l) “I had a bad experience when I got my multipurpose certificate last time. The doctors simply didn't read the reports I brought from Porto and ended up giving me a lower disability than the one I have (…). Here in [P15's area of residence] I'm worse off, already having difficulties with walking and with all the reports I've had, they gave me an incapacity rating of 79%. That is, at the moment I'm gathering the doctors' reports again so that they can assess me again and give me the disability I have and not the disability you look at. And look, 79% was, it was joking, because it's from 80% that you're entitled to the fund that they give. And I think it was, it was, they made a fool of me and I'm dealing with it now and the next time I go there I'm going to make them read the reports that they just… the reports they didn't even take out of the envelope. It was disrespectful to me.” (P15, 43, M, affected by FAP) m) “(…) Especially for younger people who are still of working age and need to keep more active, right? And from testimonies that I’ve seen at meetings of the association, I know that this limits a lot [not having access to the necessary therapies], because if people want to do physiotherapy, it’s something that… to have other therapies, right? Speech therapy and Occupational Therapies, to be effective, must be done very regularly, over a long period of time, and what is reimbursed is not in line with those needs, is it? With this type of illness, which is a chronic illness.” (P4, 50, F, Not Affected, daughter and granddaughter of individuals affected by Huntington disease)
Health Services	n)“Sometimes I think that the system itself is not build for the chronically ill, like me, I’m a chronically ill. They don’t care so much about the chronically ill, they want, they are waiting for the patients to die and when they die, that’s it, it’s one less person to bother.” (P1, 49, M, affected by Friedreich’s Ataxia) o)“Hospitals aren't… maybe they're not as prepared to deal with these diseases and to know… my father waited a long time before they were able to diagnose him. So it was very difficult in that sense. And then also, maybe, I don't know. Maybe there was little preparation for that… I can't explain it, but I think that maybe there was little, little preparation for my father's specific disease.” (P17, 25, F, daughter of a person affected by Huntington Disease) p) “Occasionally, we had to go to the emergency hospital, maybe twice. I don't have very good memories of that. The emergency services are not at all prepared for patients with the pathology he had. The professionals aren't always willing to listen to the family, who knows the patient well and their needs. (…) Once, from a rehabilitation nurse, (…) I heard tell him [P16’s husband] to move his arms, after I had explained to her that he couldn’t move his arms or legs [due to the hereditary condition] and she said, “I’m waiting for you to lift your arms” [in a rude way].” (P16, 55, F, Not Affected, wife of a person affected by ALS)
Insurance	q) “Unfortunately, I can’t share much because this is a disease that is still very… How can I explain it to you? It’s seen in a complicated way. For example, if too many people know that I have this disease – let’s say at work – I’ve told a few closer colleagues, but I don’t tell more people. Why? Because it could harm me professionally. I can’t tell too many people because if someone has bad intentions towards me, my home insurance could turn against me. These are the kinds of things that I think are unfair, but there’s nothing we can do to change them. (…) The house insurance part is the most worrying part, of course, because if I get sick, I can imagine tomorrow. I’m going to do it, not to pay, to stay, I’m going because I have an incapacity, right? They are going to know when I knew about it, how long I’ve known about it, or when I don’t know what, or when, or… and I could be harmed. It’s just those two questions.” (P3, 42, F, Asymptomatic Carrier of Huntington disease)

In family, participants emphasised discrimination experiences through withdrawal, rejection and humiliation by family members without the hereditary condition, namely by in-laws, brothers-in-laws, cousins and nephews. In the excerpts a) and b) we can see the rejection and exclusion from family meetings in the presence of other people, and in c) the humiliation against people with the hereditary condition within the family context is present.

In social context, reports of stigmatisation emerged by strangers, acquaintances and friends, mostly in public places, such as the street, the church, the municipal swimming pools and beaches. Stigmatisation manifests in the form of labels, stereotypes and discrimination through social rejection, withdrawal, stares and comments. Excerpt d) describes an experience of stigmatisation by strangers in a public place, directed at the patient and their family through the attribution of labels and stereotypes. Excerpt e) describes rejection in church. And in excerpt f) the participant, who is affected by psoriasis (a condition that leaves marks on the skin), says that she felt stigmatised particularly through stares in swimming pools where she competed and on beaches, as well as describing certain episodes in which she was forced to leave the pool.

In the school context reports emerged of physical violence and rejection from peers and exaggerated concern from teachers and staff, which were felt as a marker that accentuated a perception of difference. In excerpt g) the participant mentioned episodes in which her daughter was stigmatised by her classmates, not only because she was affected by the hereditary condition, but also because she received more attention from teachers and staff because of it. In excerpt h) the child also felt that teachers treated him differently because of his hereditary condition. In extract i) the participant mentions that her child was rejected and ignored by his peers at play.

Regarding the employment context, it was described reports of perceived stigmatisation by hierarchical superiors, through rejection in job hiring; or by colleagues, for their lack of understanding of the situation leading to the formation of labels. In this context, exclusion on the admission to employment may also result in loss of status. In excerpt j) the participant suggests that when she shared her clinical situation when looking for a job, she was often ignored, which led her to give up. In excerpt k) the participant describes situations in which her brother with Huntington's disease was labelled by colleagues because of the characteristic symptoms of the disease.

Perceived stigmatisation in social security is mostly associated with reduced accessibility to healthcare, both in terms of the difficulty in obtaining documents (e.g. medical certificate of multipurpose incapacity), as well as difficulties in granting disability benefits and access to necessary therapies. In excerpt l), the participant describes how he felt injusticed by the treatment he received from the social security doctor when he went to treat his medical certificate of multipurpose incapacity. Also in regard to social security, the reimbursement of therapies does not seem to be adequate to the reality of living with a chronic condition, which tends to increase the difficulties faced by people with hereditary conditions in their daily life and thus accentuate their perception of difference from others, as the participant describes in excerpt m).

Participants seem to notice differences when it comes to acute illnesses compared to chronic illnesses, not only regarding reimbursement of treatments by social security, but also in the access to them by health services. In excerpt n), the participant describes that little importance is given to chronic illnesses in the health services. This lack of importance, on the part of those who should be providing the greatest support for people living with hereditary conditions, can lead to a bigger feeling of difference for people living with them. In addition, the participants also mentioned the lack of preparation of hospitals to receive this type of conditions [excerpt o) and p)] and that some doctors and nurses treat patients and their families with coldness and little empathy, not considering the information that the family caregiver can provide, as exemplified in excerpt p). These excerpts suggest still a lack of knowledge and information of health professionals about the hereditary conditions and lack of specific training.

In insurance, stigmatisation manifests itself through a loss of status, particularly economic, related to life and home insurances. In order to obtain insurance, several documents and medical exams are required by the companies. The participant in excerpt q) didn't disclose her diagnosis when she took out a home insurance for fear that it would be refused, and she now lives in fear that they will find out about her condition and increase the insurance fee or even cancel it. These situations can lead to a loss of status for people living with these conditions, because if they reveal their condition, they probably won't be eligible for life, home or health insurance.

## Psychosocial impacts of stigma

This category describes the influence of real stigma, perceived, anticipated or internalized stigma on the lives of stigmatized individuals, either internally (psychological impact) or at an external level (social impact). In Table 5 we present several excerpts that support each subcategory of the psychosocial impacts of stigma.

**Table 5:** Supporting quotes for psychosocial impacts of stigma

Subcategories	Excerpts
Psychological Impacts	a) “I felt now this in my child, with teachers. Teachers, there are two or three that treat him as a “poor little guy”, and he doesn’t like it. He comes home very angry” (P6, 41, F, Affected by Skeletal Dysplasia and mother of a child affected) b) "There were people I remember, one person, a girl, who had gone to school with me and then lost contact with me because she was working abroad. I knew her family well, her parents and her sister. I worked a lot with the sister. I had a lot of contact with her sister, because we were both part of a project. And I remember when I met her [the one who went to school with P1]… I mean, I saw the parents and the sister first. They greeted me, they greeted me normally, with kisses, well, normally. She came up to me and said, ‘Hello, how are you?’ and didn't say anything else, no compliments, nothing. (…) I: And how did you feel about that? P1: I mean, on the one hand I was furious with them [people who stopped acting normally when they realised P1 had the condition], but on the other hand I also understood. On the one hand, I understood. I was furious and hated them, but of course I also tried to put myself in their shoes. I tried to put myself in their place. On the one hand I realised why they were having that reaction, but I was also frustrated, and when it's like that there's no point in getting frustrated" (P1, 49, M, affected by Friedreich’s Ataxia) c) “Unfortunately, I can’t share too much, because this is a disease which is still very… how can I explain to you, seen in a complicated way, because, for example, if many people know that I have a disease… For example, in my work, I already said to some closer colleagues, but I don’t say more, why? Because it can harm my professional career.” (P3, 42, F, Asymptomatic Carrier of Huntington disease) d) "I: And do you have any idea what it was like for him to share with other people that he had this condition? P2: Very, very complicated, very difficult, especially with his closest family. It's very complicated. (…) It started with a certain reserve in publicising it, because these things are still a bit… socially, they still have a bit of a negative weight, don't they?" (P2, 58, F, Not affected, family member of an affected person by Charlevoix-Saguenay Ataxia) e) "Most of them [schoolmates] wouldn't even call her [P10 ‘s daughter] to their birthday parties and so she felt very sad and very marginalised by her classmates" (P10, 55, M, Not Affected, father of someone affected with CCHS) f) "I think it's one of the diseases that doctors, some doctors, still don't value. And I think it's a disease that has an emotional impact that people don't realise. Myself, it's not my husband, it's not my husband who rejects me… Sometimes it's me who withdraws. It has a very big impact on me, and I don't have many signs." (P5, 53, F, affected by Neurofibromatosis and mother of two affected children) g) “I: And regarding friendship and love relationships, do you think the disease has any impact?P12: It is. I see it in my brother’s situation [affected by Huntington disease], which was an extremely social person, extremely social and always with strongly held opinions, but always very polite. And this is the reason he really had a lot of friends (…). And now he, even known he has a disease and the concern of not overdoing it or physically not going to sporting events and playing ball, for example, that he liked very much, this will also be pushing people away. And then the emotional neglect, lack of self-respect also can be seen in the way he dresses, in the way he is, in the way he manages his schedule, the places where he goes. A little bit of shame. All this isolates the Huntington’s patient, isn’t it? I don’t know” (P12, 38, F, Not Affected, daughter and sister of affected persons by Huntington Disease) h) "And I get so worried about always asking for help, because I can't do some things. Asking for help all the time, people think it's so easy, but it's not." (P1, 49, M, affected by Friedreich’s Ataxia)
Social Impacts	i) “On a love level, let’s say that, I mean let’s say, I’m a little close for this kind of situations, because it’s hard a person goes looking for another person and is in this state and that’s it, in this state of illness [locomotion difficulties], to find, let’s say, I like people, but on a love level that’s closed to me. That’s closed. I’m not going to cause any type of suffering to another person and look, I try to live with myself as best I can.” (P15, 43, M, affected by FAP) j) "When we know this [that the condition is present], we immediately start to think, "nobody is going to accept us with this pathology, nobody is going to put up with this situation" and people move away. I'm not talking in terms of friendships, but in terms of romantic relationships" (P3, 42, F, Asymptomatic Carrier of Huntington disease) k) "I'm 25 years old at the moment and no, I don't want to have relationships. I don't want to have relationships or children, because I still don't know if I have the disease or not, that's going to depend a lot on what my diagnosis is going to be, really. And I think this [not knowing the diagnosis] affects me much more in this area of not giving myself over to relationships because of this, because of the unknown that is my future." (P17, 25, F, daughter of a person affected by Huntington Disease) l) “P8: Often we realize that people were surprised or found some of her behaviour strange [mother of P8, affected by Huntington Disease], but I never had any specific situation of discrimination. Just like that, some surprise, those stares, things that, unfortunately, people still can’t control very well sometimes I: And do you think she also noticed this, she also felt that? P8: No doubt, yes, she felt that way. And, no doubts, this also discouraged her, in a way, from leaving home and being involved in society” (P8, 24, F, Not Affected, daughter of a person affected by Huntington Disease) m) "I don't like leaving here [his residence] unless I have some kind of security because of some symptoms that may occur and make me feel ashamed and embarrassed. That's it, I've been conditioned. I enjoy as much as I can. But now one of the big companies is, let's say, television. Some series, some films that I watch. And I try to pass the time that way. [by the symptoms of the condition]." (P15, 43, M, affected by FAP)

At psychological level, stigma seems to arouse emotional reactions in participants such as, frustration, anger, shame, fear of sharing that they have the disease, sadness, worry about “being a burden”, and discouragement. Frustration and anger appear for different reasons, but all highlight a perception of being treated differently by others. For example, in excerpt a) frustration and anger are related to being treated differently (even better) by teachers and in excerpt b) they are related to a notable change in the way an old friend treats the participant because of her illness. Some participants also reported a great difficulty in sharing that they have a hereditary condition, for fear that others will start treating them differently, and the fear of suffering negative consequences of exposing their hereditary condition (anticipated stigma). The participant in excerpt c) mentioned her fear of other people finding out that she has a hereditary condition, because she believes it could bring her adverse consequences on a professional level. In excerpt d) there also seems to be a fear of sharing that the disease is present because of its “negative social weight”, as the participant put it. The sadness that comes from constant rejection is reported in excerpt e) by a participant who has seen his daughter go through this. Other participants reveal shame about the symptoms of hereditary conditions. In excerpt f) the participant reveals that she sometimes distances herself from her husband because of the marks of the disease on her body, which also shows a certain internalised stigma. In extract g) the shame associated with the symptoms of a hereditary condition also translates into discouragement in carrying out activities that previously aroused interest and a lack of self-respect. Some participants also mention the fear of “being a burden”, either in a more general way as in excerpt h), where the participant reveals great concern about having to bother others because she can't do certain tasks, or in a more targeted way as in excerpt i), where the participant suggests that he doesn't allows himself to have romantic relationships because he doesn't want to be a burden (Table 5).

In addition to emotional impacts, stigma also seems to influence participants social life and interpersonal relationships, as in the excerpt mentioned above. Several participants mentioned disinvestment in relationships. In excerpt i) as already mentioned, because the participant doesn't want to be a burden in the future; in excerpt j) because of the belief that no one will want her with a hereditary condition; and in excerpt k) because of the uncertainty of her diagnosis. In the last of these, the participant also mentions her indecision about having children due to the possible presence of the disease. Isolation as consequence of stigma and at same time the withdrawal from others, as well as feelings of loneliness are also described. In excerpt l) we realise how the stares discourage them from socializing with other people and in excerpt m) how the shame of the symptoms leads the person to stay at home and take refuge in television to combat the possible loneliness.

## Coping strategies to deal with the stigma

This category describes diverse ways that participants use to manage with stigma. In Table 6 we present several excerpts for each strategy that participants mentioned to deal with stigma. Several participants refer that the best way they have to handle with stigma is to confront the stigmatisers and inform them about the hereditary condition. In the excerpt a) one participant diagnosed with Skeletal Dysplasia, an inherited condition characterised by the abnormal development of bones and cartilage that can cause limb deformities and mobility difficulties, referred that when her son, affected with the same condition, became aware of other people’s stares and comments, he began to feel different, and he isolated himself. In this way, she had to explain to her son why people acted the way they did, and later her son began to confront children and adults who look at him or commented on his body. In excerpts b) and c) the participants even take the initiative to explain to people what their illness is about. These excerpts suggest that the explanation of the hereditary condition appears as a form of adaptation in both directions. At the same time, it keeps the stigmatisers informed, it also seems to encourage patients to accept their inherited condition and consolidate their entity as a person with a hereditary condition.

**Table 6:** Supporting quotes for coping strategies to deal with the stigma

Subcategories	Excerpts
Confrontation/Explanation of the hereditary condition	a) “So, I started talking to him [son] and he began to accept it, began. Even then, sometimes people would look him, mostly the kids who started looking for him, of course children are always worse, aren’t they? And sometimes children started to say to their parents “Oh, look, this boy has legs, look, look, look how he has his legs”, “look, he has crooked legs”, “Look, he is so small”. And he turned and said “Look, do you want me to explain why I’m like this?”. And then, well, he started to become more confident, but now it doesn’t bother him at all, nor any problem.” (P6, 41, F, affected by Skeletal Dysplasia and mother of an affected person) b) "But fortunately, over time I've coped naturally and I'm the first to explain what it is, that it's not contagious. Don't worry, I'm sure you know someone who has it. There are lots of people with this disease. Always trying to get people to know more and more about the disease and to know as much about it as possible." (P11, 38, F, Affected by Hereditary Psoriasis) c) “The symptoms are a bit socially confused with a person who's drunk, or a person who's very destroyed, or a person who's careless. So, we're on the inside, we realise and we know that it's not like that, isn't it? We know that the person has a disease, so we always like to share and explain to people: what the disease is, what the symptoms are and why, the reason for the behaviour.” (P12, 38, F, Not Affected, daughter and sister of individuals affected by Huntington’s disease)
Social Isolation	d) "I: So, you feel that you never stopped doing anything because you were afraid of what other people might think or say or do? P16: No, not on my part. But my husband, I noticed that he didn't like exposing himself. I mean, I'd often say to him, ‘let's go for a walk, have some sun", etc. We had to go with the ventilator, it was fine, the chair was adapted for that, but he didn't want to go. And I, I have to be very honest, I understand, because if I see a person in a wheelchair in the street with a ventilator on, I'm going to look. Because it's not common, that's the truth, we don't see people like that in the street, right? I realise that it can be uncomfortable for the patient. If he was just in a wheelchair, without a ventilator—I see a lot of people in wheelchairs… now with a ventilator, which is a ventilator that takes up a large part of his face, I understand that he wanted to protect himself. For my part, there was no problem, I've never had a problem." (P16, 55, F, Not Affected, wife of a person affected by ALS) e) "I: Do you think she still isolated herself because she had the condition? P8: Absolutely. Yes, because people often didn't understand certain behaviours, for example, her lack of motor coordination. So obviously this had a social impact on her. She ended up isolating herself a lot more. In addition, having to deal with feelings, with other issues such as depression, made her more isolated." (P8, 24, F, Not Affected, daughter of a person affected by Huntington Disease)
Hiding/Lying about the origin of the hereditary condition	f) "I used to ask my parents not to tell people what psoriasis was because nobody knew what it was. I asked them to say it was an allergy instead." (P11, 38, F, Affected by Hereditary Psoriasis) g) “I: When did you find, how was it for you to share with other people that you have the disease? P7: I was hiding for 5 years. Just the family, my husband and my children and my sisters knew, just closer family, because my symptoms were just my eyes. And then I think at 55 years old, I couldn’t hide it anymore because I started having imbalances. Then, I told my family I: ok… and how did it feel not to share with other people? Why did you preferred to do that? P7: I don’t know to explain, I didn’t want that people know.” (P7, 61, F, affected by Machado-Joseph disease)
Humour	h) “Sometimes we even joke about it. In other words, sometimes, depending, right? Depending on the case, (…) sometimes, well, we might say, ‘Hey, maybe you’ve had a few drinks’ [one of the characteristics of the hereditary condition is stumbling], things like this that we say, but be careful, these are things that we are joking because we know that the other person is also joking about it, I’m just giving an example” (P2, 58, F, Not affected, family member of an affected person by Charlevoix-Saguenay Ataxia)
Writing	i) “I get very frustrated… what do I do to release my frustrations? I write, in my specific case, I write short stories. And it is through the short stories that I release my frustrations. (…) For me, I have already said, and I’ll say it again, for me, writing is like a catharsis. For me writing means this. It’s this that what sometimes keeps me sane.” (P1, 49, M, Affected by Friedreich’s Ataxia)
Sport	j) "Canoeing came at the right time for my son. At a time when he was having "the whys". ‘Why am I different?’, “Why am I like this?” “I don't accept myself like this”. And from the moment he joined canoeing, my son never had any more questions, it radically changed his life. And so, I became associated with them, because I think that a sport can change the life of a child who perhaps feels a little different." (P6, 41, F, Affected by Skeletal Dysplasia and mother of a child affected)

On the other hand, other participants resort to isolation as a way of dealing with the stigma. In excerpt d) the wife herself mentions how ‘different’ it is to have to go out with a ventilator in addition to her wheelchair, and understands her husband's isolation due to the stares and potential comments about the situation. In excerpt e) there is a clear response that the participant's mother isolates herself because people don't understand her behaviour.

Other participants prefer to lie about the origin of the hereditary condition [as in excerpt f)] or hide it as long as possible [as in excerpt g)], because they feel ashamed and afraid of future stigma.

There are also participants who seem to resort to humour to deal with stigma related to the hereditary condition, as we can notice in excerpt h). Writing and sport also appear as a way of confronting the stigma. In the excerpt i) one participant explains what writing means to her when she feels stigmatized, and in excerpt j) the participant explains how sport was a great way for her son to deal with stigma.

## Discussion

This is the first study that reports stigmatisation experiences of people with a group of hereditary conditions and their families in Portugal. The main results suggest that: (i) stigmatisation can be found in familiar, social, school, employment, health systems, social security and in insurance contexts; (ii) stigmatisation experiences trigger frustration, anger, sadness, shame, discouragement, fear of sharing that they have a hereditary condition and worry about “being a burden”, on a psychological level; and, on a social level, isolation, withdrawal from colleagues, friends and family and disinvestment in love relationship; (iii) individuals have different ways to cope with stigma impacts, some face and explains the condition to stigmatisers, while others isolate themselves, hide and lie about the inherited condition origin, and others resort to humour, writing and sport.

Several international studies corroborate the presence of stigmatisation in different contexts (Baynam et al. 2024; Bombard et al. 2007; Estrada-Hernandez 2018; Foji et al. 2021; Henderson et al. 2009; Williams et al. 2010). Similarly to the study by Williams et al. (2010), which includes participants from Australia, Canada and United Status of America (USA), the present study pointed to experiences of stigmatisation within the family context through rejection, withdrawal and humiliation. In social context, the aforementioned study (Williams et al. 2010) and the literature review article by Baynam et al. (2024) reports that discrimination, withdrawal and rejection by strangers, acquaintances and even friends, data that are consistent with the present study. Regarding the school context, were described, in this study, episodes of physical violence, rejection by peers and extreme concern from teachers and staff, perceived as a marker of difference. The behaviour of peers is in line with the study’s results by Estrada-Hernandez (2018), conducted in Puerto Rico, where participants affected with Albinism, described being labelled and teased by their peers, and with Henderson et al. (2009) study, where Californian participants affected by Niemann-Pick Type B disease, reported difficulties in making friends and participating in events. Foji et al. (2021), which studied the experience of living with Neurofibromatosis in many provinces of Iran, highlighted in their results the difficulty in finding a job and reported that they had been fired from their jobs due to the physical limitations of the disease and the observable characteristics of the condition. In our study, in the employment context, the participants felt and believed that they were not chosen for jobs because of the presence of the condition. Our results further corroborate the results of Bombard et al. (2007) in relation to misunderstanding of work colleagues concerning the experience of Huntington disease in Canada. In the present study, social security seemed to emphasize the difference felt by the participants throughout the lack of response regarding the acquisition of certificates and monetary funds to which they are entitled and the reimbursement of necessary therapies. The same happened with healthcare services, the perceived insensitive treatment of both patients and their families by doctors and nurses was felt as a marker of difference, in particular, compared to the treatment of acute situations. The reduced accessibility to health services, as well as discrimination felt within them, were also highlighted in Silva et al. (2013) study, in Brazilian context in participants with Sickle Cell Anaemia. Regarding insurance companies, in the study by Williams et al. (2010), the results indicate life insurance denied due to the presence of a hereditary condition, that may explain the participants fear in this study of being harmed by the existence of a hereditary condition, as reported in Bombard et al. (2007) study, from Canada.

Also, the psychological impacts associated with stigma found in this study are consistent with the literature: frustration, anger and sadness (Fournier et al. 2023), shame (Baynam et al. 2024; Fournier et al. 2023), discouragement (Williams et al. 2010), the fear of sharing they have a hereditary condition (Bombard et al. 2007) and concern about “being a burden” (Estrada-Hernandez 2018). In social context, stigma experiences seemed to generate isolation, but also withdrawal from colleagues, friends and family. Foji et al. (2021) mentioned the disinvestment in love relationships due to the belief that no one will want a person with a hereditary condition (Neurofibromatosis), also observed in our results. In addition, the present study suggests as a reason for disinvestment in love relationships the concern about “being a burden” and the participants knowledge of other stories of couples who have broken up due to the presence of a hereditary condition.

In Bombard et al. (2007) study, Canadian participants faced and explained characteristics and symptoms of Huntington disease to stigmatisers as a way of protecting themselves. In present study, these were also the ways more used by participants to cope with stigma, alongside social isolation. Isolation, due to rejection fear and to avoid hearing discriminatory comments also arises as a strategy to cope with stigma in Foji et al. (2021) study with participants affected by neurofibromatosis. In our study other participants choose to try to hide the hereditary condition and lie about their origin. The strategy of hiding the hereditary condition as much as possible or sharing the diagnosis only with close family members was also documented in the Bombard et al. (2007) and Williams et al. (2010) studies, both with participants affected or at risk of inheriting the Huntington disease. Other strategies reported in the current study, that seemed to improve people well-being regarding stigmatisation experiences, included writing, humour and sport.

In summary, these results suggest that living with a hereditary condition has implications which make psychosocial adaptation difficult for those affected and their families, with social isolation standing out. The presence of a hereditary condition in a family already brings several challenges of high complexity on its own: frequent role changes, particularly due to loss of autonomy and consequent dependence on (in)formal care; adaptation to the condition, in particular, when it manifests itself at a younger age; communication about the hereditary condition with family; among others (Street and Soldan 1998). All these challenges have impacts on the family life cycle and on family dynamics (Rolland 2018). When a disease arises in the family, this tends to turn inwards, and despite the support the family can give each other in this phase of heightened fragility, this centripetal movement can immediately reinforce the isolation of the family and the individual with a hereditary condition and increase their suffering (Rolland 2018). Besides this, when a inherited condition involves informal care, the responsible member often must quit their job and doesn’t have much time to devote to social gatherings, which is why people in their social support gradually distance themselves. Naturally, these changes in family life bring a lot of fear, worry and sadness. Our study suggests that these families also face social difficulties amplified in various contexts, which reinforce isolation. Rejection, exclusion, the stares, comments, the labelling that people living with a hereditary condition receive make them feel different, frustrated and ashamed, which leads them to develop ways of dealing with stigmatisation, namely through isolation. In other words, the exclusion of other people leads to their own exclusion, as if it were a “vicious cycle of isolation”. This isolation is associated with intense emotions of fear, sadness and shame, which in many cases translates into deep psychological suffering. In this regard, it is important to work with these families to help them integrate these experiences and “re-signify” the experience of living with a hereditary condition, for example, through family therapy (Dane et al. 2024; Zarei and Roohafza 2018), multifamily therapy (Guerra et al. 2023) or group therapy (Dane et al. 2024). Multifamily and group therapies seem very suitable for counteracting isolation, since this allows families to explore realities like their own, which is essential to normalise their experiences, emotions and thoughts (Dane et al. 2024; Guerra et al. 2023). Moreover, membership in support associations for patients and families with inherited conditions can also be a good way to counteract the difference felt. Associations seem to have a positive impact on the lives of those living with a hereditary condition, since by mobilising meaningful relationships between members, associations reduce isolation and, more importantly, seek opportunities to integrate patients into the community (Costa et al. 2022). Therefore, it is possible to rebuild identity and find more positive strategies for coping with stigma, such as education about hereditary conditions.

## Limitations

The present study has some limitations. Firstly, the exploratory nature of the study and the small number of participants do not allow the results to be generalised, so the results presented here should be analysed in future investigations with a large number of participants. Along with this, the study included a high number of autosomal dominant inherited conditions compared to other modes of inheritance, which may also result in differences in the experiences of stigma. Secondly, as the participants are volunteers, it can be inferred that they represent more positive cases of adaptation to the hereditary condition, since there were people who, when the associations publicised the study, shared that they were not comfortable sharing their experiences. In contrast, belonging to associations can mitigate the experiences of stigma, due to the sense of unity and resilience they foster. In this way, the results of the study may not broadly reflect all experiences of stigma in this population.

## Implications for practice

This study described some of the impacts of living with a hereditary condition. In most contexts where stigmatisation occurs (i.e. social, school, employment), the lack of knowledge about hereditary conditions features seems to be one of the reasons for stigmatisation, which suggests that the dissemination of information about hereditary conditions and awareness of the psychosocial impacts, through the social media, talks in the community, schools and workplaces would be beneficial. As far as health is concerned it would be beneficial to create broader treatment reimbursement measures and considering the characteristics of the individual and the hereditary condition, aiming to reduce the feeling of difference.

Offering specific training to health professionals could also be important to promote recognition of their potentially active role in activating and containing stigma. Furthermore, the investment in promoting the mental health of participants and their families seems crucial, given all the psychosocial impacts that not only stigma, but hereditary conditions itself bring.

In addition, the study warns of the importance of informing those affected and at risk of a hereditary condition about their rights, namely, the non-mandatory sharing of genetic test results, in particular with insurances and at work, as stipulated by Portuguese law 12/2005, in articles 12, 13 e 14 (República Portuguesa 2005). The same law applies in other countries, such as USA where it is known as the “Genetic Information Nondiscrimination Act” (Zukerman 2009) and in German (Manz 2016).

## Conclusion

To our knowledge, no other study describes the experiences of stigmatisation of people with hereditary conditions and their families so broadly in Portugal. People with hereditary conditions and their family members reported experiences of stigmatisation in various contexts, such as academic, work, health care, social security and insurance, highlighting the lack of knowledge in society about the hereditary conditions and its challenges. This reinforces the importance of promoting awareness in society about these conditions and their impacts. Social media, which is currently the ways in which people obtain the most information, would be a good way of making them more visible. In addition, organising talks, workshops and dynamic activities to try to show people what it's like to live with a hereditary condition, both in the community and in schools, could also be beneficial in combating stigma. Finally, reinforcing the specific formation of health professionals about hereditary conditions is also essential, as is facilitating access to healthcare.

## Data Availability

No datasets were generated or analysed during the current study.
